# Socioeconomic environment and cancer incidence: a French population-based study in Normandy

**DOI:** 10.1186/1471-2407-14-87

**Published:** 2014-02-13

**Authors:** Josephine Bryere, Olivier Dejardin, Veronique Bouvier, Marc Colonna, Anne-Valérie Guizard, Xavier Troussard, Carole Pornet, Françoise Galateau-Salle, Simona Bara, Ludivine Launay, Lydia Guittet, Guy Launoy

**Affiliations:** 1 U1086 INSERM Cancers & Preventions, Avenue du Général Harris, Caen 14076, France; 2 CHU, Avenue de la Côte de Nacre, Caen 14000, France; 3 Isere cancer registry, CHU, Grenoble, France; 4 CRLCC, Avenue du Général Harris, Caen 14076, France; 5 Public hospital, rue Trottebec, Cherbourg 50100, France; 6 Federation of cancer registries of Basse-Normandie, Caen, France

**Keywords:** Cancer incidence, Socioeconomic inequalities, Registries, Population attributable fraction

## Abstract

**Background:**

The struggle against social inequalities is a priority for many international organizations. The objective of the study was to quantify the cancer burden related to social deprivation by identifying the cancer sites linked to socioeconomic status and measuring the proportion of cases associated with social deprivation.

**Methods:**

The study population comprised 68 967 cases of cancer diagnosed between 1997 and 2009 in Normandy and collected by the local registries. The social environment was assessed at an aggregated level using the European Deprivation Index (EDI). The association between incidence and socioeconomic status was assessed by a Bayesian Poisson model and the excess of cases was calculated with the Population Attributable Fraction (PAF).

**Results:**

For lung, lips-mouth-pharynx and unknown primary sites, a higher incidence in deprived was observed for both sexes. The same trend was observed in males for bladder, liver, esophagus, larynx, central nervous system and gall-bladder and in females for cervix uteri. The largest part of the incidence associated with deprivation was found for cancer of gall-bladder (30.1%), lips-mouth-pharynx (26.0%), larynx (23.2%) and esophagus (19.6%) in males and for unknown primary sites (18.0%) and lips-mouth-pharynx (12.7%) in females. For prostate cancer and melanoma in males, the sites where incidence increased with affluence, the part associated with affluence was respectively 9.6% and 14.0%.

**Conclusions:**

Beyond identifying cancer sites the most associated with social deprivation, this kind of study points to health care policies that could be undertaken to reduce social inequalities.

## Background

Cancer is one of the leading causes of mortality worldwide and the second in the developed countries. It is thought to be responsible for around 13% of the total number of deaths, approximately 7.6 million persons dying from cancer in 2008. While cancer survival continues to improve essentially thanks to progress in treating patients and to screening, the observations concerning incidence are much less encouraging. Social deprivation can be singled out as responsible for part of this cancer incidence and the struggle against social inequalities in cancer constitutes a priority for international organizations [1].

Public action to reduce this gradient must rely in part on the proper assessment of the burden of cancer associated with social environment and on the knowledge of the mechanisms underlying such inequalities.

Studies of this type have initially focused on mortality data [2,3]. But it is important to differentiate between social disparities in incidence of cancer and social disparities in survival as it was the case in the literature of the recent years. The relationship between cancer incidence and socioeconomic status is dynamic and needs to be continuously monitored.

The mechanisms by which the social environment influences the risk of cancer are many and varied. None of these mechanisms are exclusive and all interact. Based on the work of previous authors, these mechanisms are organized in behavioral models focusing on individual determinants [4,5] (alcohol, tobacco, diet, physical exercise, practice prevention, etc.), or contextual models focusing on complexity determinants [6,7] (occupational exposure, general exposure, access to health system, etc.). This complexity suggests that a proper evaluation of the social environment should not be limited to any particular indicator such as financial resources, education or profession, but should appreciate the social environment in its entire individual and collective dimension. Geographical approaches are thus particularly relevant for studying the link between social environment and cancer incidence. Moreover, from a public health point of view, the measure of the human cost of these inequalities at an aggregated level is particularly relevant for potential further actions.

The objective of the study was to quantify the part of the cancer burden related to social deprivation. We firstly identified the cancer sites linked to the socioeconomic status of the living area and secondly measured for each one the proportion of cases of cancer associated with social deprivation.

## Methods

### Study population

The population comprised all cases of cancer diagnosed in Calvados and Manche, two French *departements* in *Basse*-*Normandie*, from 1997 to 2009 and recorded in the five local registries: Calvados cancer registry, digestive Calvados registry, Manche cancer registry, Malignant hematological *Basse*-*Normandie* registry and Multicentral mesothelioma registry. The whole population comprised 68 967 cases divided into 29 cancer sites (Table 1). According from INSEE (Institut National de la Statistique et des Etudes Economiques), the population of Calvados and Manche is composed of 48% of men and 52% of women which is equivalent to the national distribution. The population is slightly older than the national average. In Calvados and Manche, 47% of individuals are under 40 years compared to 50% nationally, and 26% are over 60 years compared to 23% nationally. The economy is also less efficient with a GDP of 2.1%; it stands at 3.1% nationally.

**Table 1:** Site definitions and frequencies in Normandy between 1997 and 2009

**Site**	**ICD-O-3**		**Frequencies**		
	**Topography**^a^	**Morphology**^a^	**Men**	**Women**	**Total**
Prostate	C61	All	11611		11611
Breast	C50	All		10893	10893
Lung	C33, C34	All	6095	1324	7419
Colon-rectum	C18, C19, C20, C21	All	3983	3206	7189
Lips-mouth-pharynx	C0, C10, C11,	All	3153	579	3732
	C12, C13, C14	All			
Bladder	C67	All	2452	590	3042
Kidney	C64, C65, C66, C68	All	1334	737	2071
Non-Hodgkin	All	95903-95963 or	1071	945	2016
lymphoma		96703-97193 or			
		97273-97293 or			
		98323-98343			
Stomach	C16	All	1186	691	1877
Melanoma	C44	87203-87803	725	1063	1788
Unknown primary sites	C76, C809,	All	925	654	1579
Central nervous system	C70, C71, C72	≤ 91103 or ≤91800	719	801	1520
Corpus uteri	C54	All		1449	1449
Pancreas	C25	All	786	660	1446
Liver	C22	All	1148	238	1386
Esophagus	C15	All	1138	208	1346
Ovary	C56, C570, C571,	All excluding		1247	1247
	C572, C573, C576	{84423; 84513;			
		84613; 84623;			
		84723; 84733}			
Myeloma	All	97313-97343 or	646	543	1189
		97603-97643			
Thyroid	C73	All	258	884	1142
Larynx	C32	All	867	86	953
Lymphocytic leukemia	All	98233	508	409	917
Cervix uteri	C53	All		764	764
Leukemia	All	98013-98203 or	393	356	749
		98263-98273 or			
		98353-98613 or			
		98663-98743 or			
		98913-99203 or			
		99483			
Gall bladder and extrahepatic bilary tract	C23, C24	All	185	254	439
Testis	C62	All	400		400
Hodgkin’s lymphoma	All	96503-96673	209	155	364
Mesothelioma	C384	All	190	60	250
Small intestine	C17	All	98	91	197
All cancers	C00 to C80	All	40080	28887	68967

^a^Hematological codes are always excluded from solid tumor sites and included in the relevant hematological site.

### Variables

The clinical characteristics of the tumors were collected by the registries in a standardized way ensuring the completeness and good quality of the data. The site, morphology, age, gender and diagnosis date were known for every patient.

For all cases of cancer diagnosed, place of residence was geolocalized with a Geographic Information System (GIS) running on MAPINFO 10.0 and allocated to an IRIS (Ilots Regroupes pour l'Information Statistique), a geographical area defined by INSEE [8]. It is the smallest geographical unit for which census data are known, a factor essential for this kind of study [9]. There are 1496 IRIS in the two departments. The smallest IRIS is composed of 10 inhabitants, the biggest is composed of 4811 inhabitants and the mean is 755. The database provided the number of cancer cases diagnosed in an IRIS for the whole period.

The reference population came from the INSEE social census 1999 and 2006. It is given for each IRIS, each sex and each age group: [0–14], [15–29], [30–44], [45–59], [60–74], [75 and more]. The population was linearly extrapolated for the whole period 1997–2009. Knowing the population sizes for an IRIS, an age group and a gender for the years 1999 and 2006, supposing that an increase or a decrease of the sizes were constant, we extrapolated the population sizes for the years 1997, 1998, 2000, 2001, 2002, 2003, 2004, 2005, 2007, 2008, 2009.

The recently published French EDI (European Deprivation Index) was used to attribute a social deprivation score to the IRIS [10]. The methodology used an individual deprivation indicator from the conceptual definition of deprivation and selected ecological census variables that are the most closely related to the individual deprivation indicator in the European Union Statistics on Income and Living Conditions (EU-SILC). This was available as a continuous variable, increasing from - 5.33 to 20.52. Depending on the modelling performed, the continuous version of the EDI variable or a categorical version (quintiles calculated at the French level) was used.

### Statistical analysis

A Bayesian approach was used rather than the classical Poisson regression because it allows the integration of extra-Poisson variability if it exists in the data. The differences in population sizes between IRIS, called unstructured spatial heterogeneity, may have introduced variations and this methodology permits the distinction between random fluctuations and true variations in incidence rates. Moreover, neighboring areas may not be independent and have similar incidence rates and this phenomenon, called spatial autocorrelation, is also integrated with the Bayesian approach [11,12] performed using WinBUGS version 1.4 [13]. It is written as follows:

$$\log\left(y_{i}\right)=\log\left(E_{i}\right)+\alpha +\beta \;\mathrm{ED}I_{i}+V_{i}+U_{i}$$

where y_i_ and *E*_*i*_ are the observed and expected number of cases in area *i*. $E_{i}=\underset{j,k}{\sum }t_{j,k}P_{j,k}$ where *t*_*j*,*k*_ is the global incidence rate for the age group *j* and sex *k* and *P*_*j*,*k*_ is the population size for the IRIS *i*, age group *j* and sex *k*. α is the intercept, representing the global relative risk, *β* the coefficient associated with the variable *EDI*, *U*_*i*_ is the structured variation (spatially structured heterogeneity) and *V*_*i*_ is the unstructured variation (non spatially structured heterogeneity). The *EDI* coefficient was estimated with its 95% credible intervals (CIs) for each cancer site. A positive *EDI* parameter means an over-incidence in deprived areas and a negative *EDI* parameter means an over-incidence in affluent areas. We calculated exp (β) for significant sites because it reflects the excess risk related to *EDI*. Living in an IRIS with a highest deprivation score of one over another, increases the risk of developing a cancer of exp (β).

To know whether spatial autocorrelation and spatial heterogeneity were actually in the data, we first performed a Moran test [14] for autocorrelation and a Potthoff-Wintinghill test [15] for heterogeneity. They were performed with packages spdep and DCluster from R version 2.15.0, p-values of the tests being indicated in tables. If both tests were significant we performed a BYM (Besag, York and Mollié) model integrating the two components, if just the Moran test was significant we performed a CAR (Conditional Auto Regressive) model integrating the spatially structured heterogeneity, if just the Potthoff-Wintinghill test was significant we performed a model with the non-spatially structured heterogeneity and if both tests were non-significant, meaning that there was no variability of incidence in the data, the integration of EDI was not included in the analysis.

The final step was to assess for each cancer site the Population Attributable Fraction (PAF) [16,17]. It can be defined [16] as the proportional reduction in average disease risk over a specified time interval that would be achieved by eliminating the exposure of interest from the population. To do so, the national quintile version of the deprivation index *EDI* was used and included in the model. The quintiles were named *Q*_1_ to *Q*_5_, *Q*_1_ being the quintile of the least deprived group and *Q*_5_ the quintile of the most deprived one. A relative risk was determined for each social deprivation level and was called *RR*_1_ to *RR*_5_. The relative risks were calculated using the exact same model as above, except that the categorical version of the *EDI* (by quintile) was introduced into the model. If a significant and a positive beta coefficient were observed, then *Q*_1_ was considered as the reference category. If a significant and a negative beta coefficient were observed, then *Q*_5_ was considered as the reference category. The relative risk of the reference category was set to 1. The associated proportion of risk was defined as:

$$\mathrm{PAF}=1-\frac{1}{\sum_{i=1..5}p_{i}RR_{i}}$$

*P*_*i*_ is the proportion of the population at the national quintile *i*.

## Results

For the whole study period, 68 967 cases of cancer were recorded in Calvados and Manche, 40 080 men and 28 887 women.

The most frequent sites in decreasing order were prostate, breast, lung, colon-rectum and lips-mouth-pharynx (Table 1).

Concerning the continuous deprivation index *EDI*, the minimum was -3.77 for the most affluent IRIS and the maximum was 8.98 for the most deprived IRIS, the median being -0.45. Quintiles being defined at a national level, 20% of the population was situated at the first quintile, 22% at the second, 23% at the third, 23% at the fourth and 12% at the fifth.

Tables 2 and 3 present the results of modelling using the continuous version of *EDI*.

**Table 2:** Influence of socioeconomic deprivation of living area on cancer incidence in men in Normandy between 1997 and 2009

**Site**	**Moran test**	**PW test**	**Estimation**^**a**^	**CI**^**b **^**(95%)**	**Exp (β)**
	**p-value**	**p-value**	***EDI *** **coefficient**		
Prostate	0.33	< 0.05	**-0.023**	**[-0.043; -0.010]**	0.98
Lung	< 0.025	< 0.05	**0.087**	**[0.065; 0.108]**	1.09
Colon-rectum	< 0.025	< 0.05	0.025	[-0.001; 0.050]	
Lips-mouth-pharynx	< 0.025	< 0.05	**0.149**	**[0.122; 0.176]**	1.16
Bladder	< 0.025	< 0.05	**0.033**	**[0.001; 0.064]**	1.03
Kidney	<0.025	< 0.05	0.033	[-0.003; 0.069]	
Stomach	< 0.025	< 0.05	0.001	[-0.047; 0.047]	
Liver	< 0.025	< 0.05	**0.076**	**[0.039; 0.114]**	1.08
Esophagus	< 0.025	< 0.05	**0.086**	**[0.043; 0.131]**	1.09
Non-Hodgkin lymphoma	0.04	< 0.05	-0.006	[-0.047; 0.035]	
Unknown primary sites	0.11	< 0.05	**0.060**	**[0.019; 0.101]**	1.06
Larynx	< 0.025	< 0.05	**0.154**	**[0.114; 0.196]**	1.17
Pancreas	0.65	< 0.05	0.019	[-0.029; 0.066]	
Melanoma	0.86	< 0.05	**-0.078**	**[-0.132; **-**0.026]**	0.92
Central nervous system	< 0.025	< 0.05	**0.056**	**[0.010; 0.101]**	1.06
Myeloma	0.84	< 0.05	-0.024	[-0.080; 0.030]	
Lymphocytic leukemia	0.98	< 0.05	-0.040	[-0.105; 0.023]	
Testis	< 0.025	< 0.05	-0.029	[-0.094; 0.036]	
Leukemia	0.43	< 0.05	-0.013	[-0.080; 0.052]	
Thyroid	0.09	< 0.05	0.020	[-0.057; 0.095]	
Hodgkin’s lymphoma	0.16	< 0.05	-0.085	[-0.180; 0.005]	
Gall-bladder and	< 0.025	< 0.05	**0.141**	**[0.058; 0.221]**	1.15
Extrahepatic bilary tract					
Mesothelioma	< 0.025	< 0.05	0.057	[-0.172; 0.052]	
Small intestine	0.69	< 0.05	0.009	[-0.127; 0.135]	

^a^Positive for an over-incidence in deprived areas, negative otherwise.

^b^Significant CIs are in bold type.

**Table 3:** Influence of socioeconomic deprivation of living area on cancer incidence in females in Normandy between 1997 and 2009

**Site**	**Moran test**	**PW test**	**Estimation**^**a**^	**CI**^**b **^**(95%)**	**Exp (β)**
	**p-value**	**p-value**	***EDI *** **coefficient**		
Breast	< 0.025	< 0.05	-0.016	[-0.032; 0.001]	
Colon-rectum	< 0.025	< 0.05	-0.001	[-0.026; 0.026]	
Corpus uteri	< 0.025	< 0.05	0.024	[-0.011; 0.059]	
Lung	< 0.025	< 0.05	**0.075**	**[0.037; 0.113]**	1.08
Ovary	0.69	< 0.05	-0.031	[-0.069; 0.006]	
Melanoma	0.49	< 0.05	-0.028	[-0.068; 0.012]	
Non-Hodgkin lymphoma	0.79	< 0.05	-0.004	[-0.046; 0.038]	
Thyroid	< 0.025	< 0.05	0.002	[-0.043; 0.047]	
Central nervous system	< 0.025	< 0.05	0.024	[-0.044; 0.051]	
Cervix uteri	< 0.025	< 0.05	**0.094**	**[0.052; 0.136]**	1.10
Kidney	< 0.025	< 0.05	0.021	[-0.026; 0.068]	
Stomach	< 0.025	< 0.05	0.007	[-0.052; 0.067]	
Pancreas	0.05	< 0.05	0.045	[-0.004; 0.091]	
Unknown primary site	0.56	< 0.05	**0.065**	**[0.015; 0.113]**	1.08
Bladder	< 0.025	< 0.05	0.033	[-0.023; 0.086]	
Lips-mouth-pharynx	< 0.025	< 0.05	**0.103**	**[0.054; 0.150]**	1.11
Myeloma	0.35	< 0.05	-0.038	[-0.096; 0.020]	
Lymphocytic leukemia	< 0.025	< 0.05	0.041	[0.024; 0.104]	
Leukemia	0.04	< 0.05	-0.036	[-0.107; 0.034]	
Gall-Bladder and	0.93	< 0.05	-0.014	[-0.097; 0.068]	
Extrahepatic bilary tract					
Liver	< 0.025	< 0.05	0.078	[-0.002; 0.154]	
Esophagus	0.40	< 0.05	0.068	[-0.017; 0.151]	
Hodgkin’s lymphoma	0.33	0.11			
Small intestine	0.88	0.14			
Larynx	< 0.025	0.06	0.110	[-0.005; 0.217]	
Mesothelioma	< 0.025	0.05	0.040	[-0.144; 0.205]	

^a^Positive for an over-incidence in deprived areas, negative otherwise.

^b^Significant CIs are in bold type.

The Potthoff-Whittinghill test and the Moran test were significant for a majority of sites.

The link between incidence and social deprivation was not significant for a majority of cancer sites in both genders, was positive for 9 sites in males and 4 sites in females and was negative for two in males and none in females. For lung, lips-mouth-pharynx and unknown primary sites, the link was positive in both genders. We obtained similar betas for both genders but the sites concerned were more frequent in males so the impact in terms of number of cases was greater in males. The link was positive in males only for bladder, liver, esophagus, larynx, central nervous system and gall-bladder and in females only for cervix uteri. The highest relative risks concerned lips-mouth-pharynx in both genders, larynx and gall-bladder in males and cervix uteri in females.

Tables 4 and 5 present the relative risks calculated using the quintile version of *EDI* and the results of the calculation of the PAF.

**Table 4:** Analysis using the quintile version of EDI and Population Attributable Fraction in males between 1997 and 2009

**Site**		**RR**	**CI**	**PAF**^**a **^**(%)**
Prostate	Quintile 1	1.19	[1.09; 1.29]	9.6
	Quintile 2	1.13	[1.04; 1.22]	
	Quintile 3	1.04	[0.96; 1.12]	
	Quintile 4	1.15	[1.06; 1.24]	
	Quintile 5	1		
Lung	Quintile 1	1		9.9
	Quintile 2	1.07	[0.97; 1.19]	
	Quintile 3	0.99	[0.88; 1.10]	
	Quintile 4	1.18	[1.06; 1.31]	
	Quintile 5	1.44	[1.29; 1.61]	
Lips-mouth-pharynx	Quintile 1	1		26.0
	Quintile 2	1.23	[1.06; 1.43]	
	Quintile 3	1.20	[1.03; 1.39]	
	Quintile 4	1.54	[1.34; 1.78]	
	Quintile 5	2.05	[1.77; 2.05]	
Bladder	Quintile 1	1		6.0
	Quintile 2	1.10	[0.95; 1.27]	
	Quintile 3	0.93	[0.80; 1.09]	
	Quintile 4	1.51	[0.99; 1.34]	
	Quintile 5	1.19	[1.01; 1.40]	
Liver	Quintile 1	1		6.9
	Quintile 2	1.04	[0.85; 1.27]	
	Quintile 3	0.93	[0.75; 1.14]	
	Quintile 4	1.14	[0.94; 1.38]	
	Quintile 5	1.40	[1.15; 1.71]	
Esophagus	Quintile 1	1		19.6
	Quintile 2	1.30	[1.05; 1.63]	
	Quintile 3	1.17	[0.95; 1.47]	
	Quintile 4	1.24	[1.01; 1.54]	
	Quintile 5	1.67	[1.34; 2.11]	
Unknown primary sites	Quintile 1	1		9.7
	Quintile 2	0.99	[0.79; 1.26]	
	Quintile 3	1.12	[0.89; 1.41]	
	Quintile 4	1.18	[0.95; 1.47]	
	Quintile 5	1.13	[1.03; 1.65]	
Larynx	Quintile 1	1		23.2
	Quintile 2	1.05	[0.81; 1.35]	
	Quintile 3	1.24	[0.98; 1.58]	
	Quintile 4	1.54	[1.22; 1.95]	
	Quintile 5	1.91	[1.49; 2.45]	
Melanoma	Quintile 1	1.37	[1.07; 1.77]	14.0
	Quintile 2	1.16	[0.89; 1.49]	
	Quintile 3	1.06	[0.82; 1.37]	
	Quintile 4	1.18	[0.92; 1.50]	
	Quintile 5	1		
Central nervous system	Quintile 1	1		9.4
	Quintile 2	1.05	[0.81; 1.35]	
	Quintile 3	1.16	[0.91; 1.47]	
	Quintile 4	1.15	[0.90; 1.44]	
	Quintile 5	1.19	[0.93; 1.54]	
Gall-bladder	Quintile 1	1		30.1
	Quintile 2	1.59	[0.94; 2.80]	
	Quintile 3	1.32	[0.77; 2.27]	
	Quintile 4	1.31	[0.90; 2.60]	
	Quintile 5	1.88	[1.11; 3.24]	

^a^PAF calculated with quintile 1 as reference except for prostate cancer and melanoma.

**Table 5:** Analysis using the quintile version of EDI and Population Attributable Fraction in females between 1997 and 2009

**Site**		**RR**	**CI**	**PAF**^**a **^**(%)**
Lung	Quintile 1	1		9.0
	Quintile 2	1.09	[0.88; 1.35]	
	Quintile 3	1.12	[0.84; 1.29]	
	Quintile 4	1.10	[0.89; 1.35]	
	Quintile 5	1.37	[1.11; 1.71]	
Cervix uteri	Quintile 1	1		5.2
	Quintile 2	0.88	[0.67; 1.15]	
	Quintile 3	1.05	[0.81; 1.35]	
	Quintile 4	1.09	[0.86; 1.39]	
	Quintile 5	1.40	[1.10; 1.80]	
Unknown primary sites	Quintile 1	1		18.0
	Quintile 2	1.21	[0.89; 1.65]	
	Quintile 3	1.15	[0.84; 1.54]	
	Quintile 4	1.43	[1.08; 1.91]	
	Quintile 5	1.29	[0.95; 1.74]	
Lips-mouth-pharynx	Quintile 1	1		12.7
	Quintile 2	0.98	[0.72; 1.35]	
	Quintile 3	1.08	[0.78; 1.47]	
	Quintile 4	1.29	[0.96; 1.72]	
	Quintile 5	1.52	[1.11; 2.05]	

^a^PAF calculated with quintile 1 as reference.

**Figure 1:**
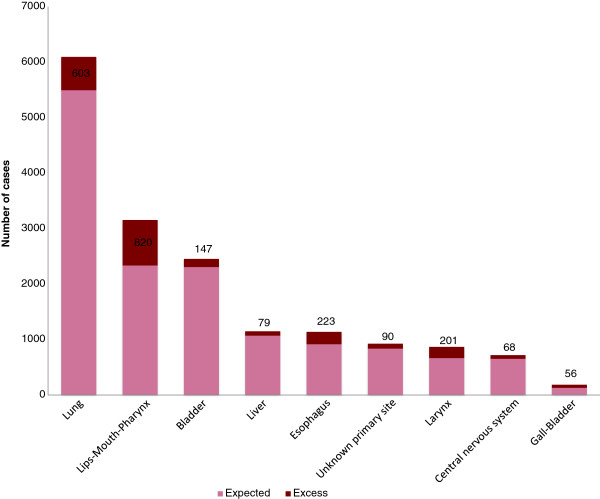
Proportion of excess cases associated with social deprivation in men.

**Figure 2:**
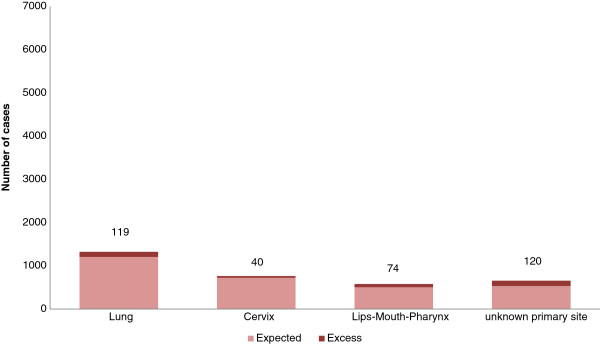
Proportion of excess cases associated with social deprivation in women.

Using the calculation of PAF, the greatest part of the incidence associated with deprivation was found for lips-mouth-pharynx cancer, esophageal cancer, laryngeal cancer and gall-bladder in males, respectively 26.0%, 19.6%, 23.2% and 30.1%. In females, the greatest part of the incidence associated with deprivation was found for unknown primary sites (18.0%) and lips-mouth-pharynx (12.7%). For prostate cancer and melanoma in males, the sites where incidence increased with affluence, the part associated with affluence was respectively 9.6% and 14.0%. The excess cases due to social deprivation are represented in Figures 1 and 2. The highest number of cases attributable to social deprivation concerned lips-mouth-pharynx cancer in males (n = 820) (Figure 1) and unknown primary sites (n = 120) (Figure 2) in females and for prostate cancer, 1115 cases can be considered as excess cases due to affluence and for melanoma in males, 90 cases can be considered as excess cases due to affluence. By adding excess cases associated with deprivation, we find 2287 excess cases in men (5.7% of the total number of cancers in men) and 353 in females (1.2% of the total number of cancer in females).

## Discussion

This study provides evidence of social disparities in the incidence of cancers. Most of these disparities consist in an over-incidence for the most deprived, especially for lips-mouth-pharynx, lung, unknown primary sites, bladder and larynx cancers. Both genders are concerned, but the impact is greater in men, considering the huge frequency of these cancer sites for them. These inequalities in incidence are all the more serious and the cancer burden is all the greater in that the cancer sites concerned are those associated with very low survival. For the period 1997–2009, analysis with the PAF showed that the social gradient generated 2287 (5.7%) excess cases in men and 353 (1.2%) in females. By analyzing site by site, the social gradient generated up to 30.1% (gall-bladder) and 18.0% (unknown primary sites) extra cases in men and women respectively.

The sites identified as linked with socioeconomic status are not surprising and consistent with previous papers. Thus, the highest incidence for lung, lips-mouth-pharynx, esophagus, larynx, bladder and liver cancer in low socioeconomic status can be explained by a higher consumption of alcohol and tobacco in the most disadvantaged [5,18,19]. Similarly, the trend in over-incidence of cervical cancer in deprived women can be explained by sexual behaviors and/or lower participation in pap smear screening [20]. The highest incidence of cancers with unknown primary sites in males and females with a low socioeconomic status can be explained by the fact that the group of “unknown primary sites” mainly comprised subjects with metastatic cancers where the primary site could not be identified, a situation more frequent in people with a low socioeconomic status [21]. Results in the literature concerning the relation between incidence of central nervous system cancer socioeconomic status are contradictory. The etiology of cerebral tumors remains unclear [22,23]. The results concerning gall-bladder are consistent with previous papers. People with a low socioeconomic status may have a diet and a feeding behavior which contribute to a development of the disease [24]. The trend in over-incidence of prostate cancer may come from the higher participation of high socioeconomic classes in screening activities and since PSA screening is associated with over diagnosis [25]. The higher participation of high socioeconomic classes in screening activities can also explain the higher incidence for affluent patients for melanoma in males and this higher incidence can also be explained by holidays abroad and exposure to natural UV [17,26]. Conversely, the absence of a social gradient in the incidence of breast seems surprising, since it is targeted by screening associated with social inequalities in participation, and because well-established risk factors such as late age at first birth or hormone replacement are more prevalent in high socioeconomic groups [6]. The spatial nature of the data and its specificities (spatial autocorrelation and non spatially structured heterogeneity) was accounted in our modelling thanks to the Bayesian approach ensuring a good consistency of the statistical analysis. Such a methodology was not integrated in previous studies treating cancer incidence and social disparities, preferring a classical Poisson regression, and thus risking to underestimate the standard error and to wrongly conclude at a significant effect of deprivation on cancer incidence [27].

Our study has several limits. By using the PAF and in absence of individual data, we sought to quantify social inequalities in incidence of cancer, rather than understand the underlying mechanisms. Using a neighborhood-based index instead of a set of individual indicators has the advantage of incorporating both individual and collective determinants that jointly mediate the social environment, but this inevitably introduces an ecological bias for appropriate measurement of individual socioeconomic status. Moreover, it considerably limits the search for causative factors explaining the links between social environment and occurrence of cancer, individual measures of socioeconomic status and behavioral risk factors being the best means to explore in more depth the mechanisms responsible for the influence of social environment on cancer risk. In addition, the social environment was measured only at the time of diagnosis, using the current address of patients but ignoring their history of mobility, which could be geographical and across social classes. Furthermore, we focused on the consequences of previous social inequalities owing to the delay between exposure and diagnosis. Despite the large number of cases analyzed from cancer registries that have a high level of case ascertainment, consistency and representativeness, a lack of power cannot be excluded for the less frequent cancer sites.

Extrapolation of the PAFs needs further investigations in order to ascertain their variability due to gradient in relative risks, or to distribution across social quintiles. Errors in interpretation can appear, as highlighted in the article by Rockhill, *et al*. [16] with the use of the PAF. Firstly, Rockhill *et al*. point out many errors possible when analyzing multiple risk factors which is not the case of our study. The second point is the overuse of the word “explain” in the interpretation of the PAF. Rather than explain, it measures the extent of the phenomenon of deprivation on cancer incidence. The PAF should be considered as the population resultant of the overall excess of cases in deprived compared with privileged people. The socioeconomic environment is not a causal factor of cancer in the biological sense of the term. However, since much of the proximal risk factor is more prevalent in the deprived, the socioeconomic environment can be considered as the "cause of the cause", a distal determinant, pathways from deprivation to health including different types of mediators such as behavioral, community, social, educational, work-related, cultural and political factors [28]. Such quantification of social disparities at a community level points to the need to jointly take actions in a universal approach and also in approaches targeting deprived people, rather than global population actions only that fail to reduce social gradients because they generally benefit the more affluent. The PAF makes it possible to estimate the collective gain that could be obtained by public actions aiming to reduce the social gradient of incidence by measuring the extent of the population for which it is necessary to lead effective cancer prevention.

## Conclusions

This study proposes an estimation of the proportion of cancers associated with social deprivation and show how by decreasing socioeconomic variation in incidence with policies aiming to reduce social inequalities, an important impact could be made on the burden of cancer.

## Competing interests

The authors declare that they have no competing interests.

## Authors’ contribution

JB, OD and GL worked on the conception and design. OD, VB, AVG, XT, FGS, SB, CP and LL participated in the acquisition of data. JB performed the analysis and interpreted the data with OD, VB, MC, LG and GL. JB, OD, VB, MC, CP, LG and GL revised the manuscript and all authors read and approved the final manuscript.

## Pre-publication history

The pre-publication history for this paper can be accessed here:

http://www.biomedcentral.com/1471-2407/14/87/prepub
